# Effect of Vanadium Oxide on the Crystallization of CaO–Al_2_O_3_–SiO_2_ Glass

**DOI:** 10.1021/acsomega.2c08246

**Published:** 2023-02-23

**Authors:** Shingo Machida, Ken-ichi Katsumata, Kei Maeda, Atsuo Yasumori

**Affiliations:** Department of Material Science and Technology, Faculty of Advanced Engineering, Tokyo University of Science, 6-3-1 Niijuku, Katsushika-ku, Tokyo 125-8585, Japan

## Abstract

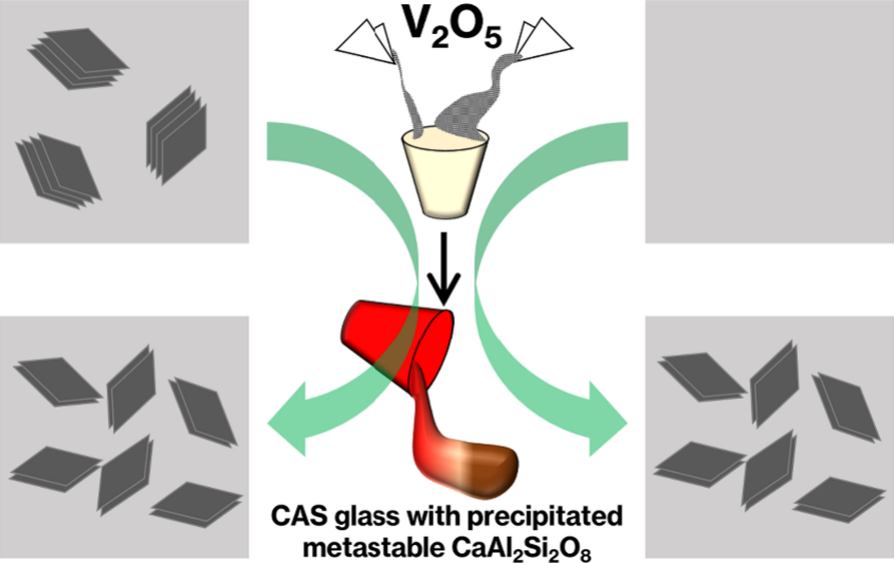

This study investigated the effect of vanadium oxide on the crystallization of CaO–Al_2_O_3_–SiO_2_ (CAS) glass. Specifically, this study subjected CAS glass-ceramics (GCs) with precipitated hexagonal platy particles of metastable CaAl_2_Si_2_O_8_ (CAS GC-H), a layered crystal, that was prepared using metallic molybdenum (Mo) particles as nucleation agents. When the parent glass of CAS GC-H was crystallized with the addition of vanadium oxide in the 0.052–0.21 wt % range, the obtained platy particles of metastable CaAl_2_Si_2_O_8_ displayed an increase in the aspect ratio from 20 to 15 compared with conventional CAS GC-Hs. In addition, no crystallization occurred in the CAS glass with vanadium oxide in the 0.052–0.21 wt % range in the absence of metallic Mo particles. Meanwhile, a CAS glass containing 1.0 wt % vanadium oxide without the addition of metallic Mo particles showed the precipitation of metastable CaAl_2_Si_2_O_8_. Therefore, these results indicated that the aspect ratio of layered crystals in glass was controlled by the addition of a relatively small content of vanadium oxide, and a new nucleation agent for the precipitation of metastable CaAl_2_Si_2_O_8_ in CAS glass using a relatively high content of vanadium oxide was developed.

## Introduction

Following the discovery of glass-ceramics (GCs), composite materials in which a crystalline phase is present within a glassy phase, which were initially prepared using silver nanoparticles as nucleation agents, GCs with varied compositions, crystals, and microstructures have been widely investigated to explore their fundamental properties for practical applications.^1−4^ Thus, nucleation agents such as titania and zirconia that dominate bulk crystallization can be essential.^1−4^ Among multiple nucleation agents, metallic Mo and W particles are the best candidates to control the microstructure of CaO–Al_2_O_3_–SiO_2_ (CAS) GCs with precipitated hexagonal platy particles of metastable CaAl_2_Si_2_O_8_ (CAS-GC-H), a layered crystal, in the 3–110 μm range.^4−9^ In addition, this particle size control was achieved by the single composition of parent glasses and the same heating conditions without byproduct formation.^4−9^ Although the volume fraction of CAS GC-H is relatively low^4−9^ and can be a drawback to separately characterize glassy and crystalline phases, the mechanical properties induced in CAS GC-Hs by the house-of-cards structure formed by layered crystals similar to those of mica were recently analyzed using X-ray computed tomography.^10^ The microstructure of CAS GC-Hs is therefore worth further controlling to elucidate the mechanism of mechanical properties. In this trial, the single crystalline phase and composition as well as the same heating condition mentioned above could be advantageous. In our previous report, a decrease in the size of platy particles in CAS GC-Hs was accomplished by melting the raw materials under an oxidizing atmosphere to decrease the size of the metallic W particles, whereas the effect of an oxidizing atmosphere was not significant for CAS GC-Hs prepared using metallic Mo particles.^7^ Despite this difference between Mo and W particles, the oxidation of metallic particles generates their oxides, suggesting the presence of both metals and their oxides in CAS GC-Hs and their parent glass.^7^ These oxides could affect the crystallization process, but this is complicated by the co-presence of metals and their oxides in the glass whose ratios could be concurrently changed during melting and heat treatments. Thus, metals such as vanadium whose oxidation free energies and melting points are lower than those of metallic W and Mo are helpful for elucidating the effect of oxides generated by the oxidation of metals in glass on the crystallization behavior.^11^ In addition, such metals could be present as oxides in glasses. Furthermore, vanadium oxides are present in glasses in a similar fashion to molybdenum oxides.^12−16^ In this study, therefore, special attention has been paid to investigating the effect of vanadium oxide on the precipitation of metastable CaAl_2_Si_2_O_8_ in CAS glass using metallic Mo.

## Experimental Section

### Materials

Calcium carbonate (CaCO_3_), aluminum oxide (Al_2_O_3_), silica (SiO_2_), and vanadium oxide (V_2_O_3_) were obtained from Wako Pure Chemical. Molybdenum oxide (MoO_3_) and carbon were obtained from Kojundo Chemical Laboratory. All chemicals were used without further purification.

### Sample Preparation

The glass specimens were prepared according to our previous reports;^5−9^ the melting conditions were 1550 °C for 1 h under air in an alumina crucible. Batches were prepared by mixing CaCO_3_, Al_2_O_3_, and SiO_2_ to form 50 g of 25CaO–20Al_2_O_3_–55SiO_2_ glass (wt %) with 0.0, 0.025, or 0.050 wt % MoO_3_, 0.08, 0.2, or 0.4 wt % C, and 0.0, 0.052, 0.21, 0.52, or 1.0 wt % V_2_O_3_. The glass specimens and the contents of MoO_3_, C, and V_2_O_3_ as well as the molar ratios of MoO_3_ to V_2_O_3_ (MoO_3_: V_2_O_3_) are given in Table 1. The glass batches were melted to obtain glass cullet and then remelted with the addition of the same weight and compositional batch. For Product-E and -F, the same compositional batch was not added. After annealing these melts at 850 °C for 30 min, the glass specimens were heat-treated at 1050 °C for 2 h. Notably, the lack of bubbles or voids were observed in both glass specimen before and after crystallization. After the heat treatment, the surface layer was removed by polishing. The specimens were cut and polished to form appropriately sized or shaped specimens for each analysis as described below.

**Table 1:** Glass Specimens and Additive Content

product names	MoO_3_ (wt %)	V_2_0_3_ (wt %)	C (wt %)	molar ratio (Mo0_3_: V_2_0_3_)
Product-A	0.050	0.052	0.4	1:1
Product-B	0.025	0	0.4	
Product-C	0.025	0	0.2	
Product-D	0.050	0.21	0.08	1:4
Product-E	0.050	0	0.08	0
Product-F	0	0.052	0.08	0
Product-G	0	0.52	0.4	
Product-H	0	1.0	0.4	

### Characterization

The crystalline phases and microstructure of the glass specimens after the 1050 °C heat treatment were assessed by powder X-ray diffraction (XRD; XRD-6100, Shimadzu) and scanning electron microscopy (SEM; TM-3000, Hitachi). The volume fractions for the heated glass specimens were approximately estimated by analysis of binarized SEM images. As in our previous studies,^4−9^ SEM images of house-of-cards structures comprising platy particles of metastable CaAl_2_Si_2_O_8_ appeared as black regions with needle-like particles that represent an arbitrary cross-section of platy particles in a house-of-cards structure. We thus tentatively denoted the needle-like particles as platy particles of metastable CaAl_2_Si_2_O_8_ whose size was defined as the longitudinal length, the lateral size of platy particles.^6−8^ The aspect ratio^17,18^ is also defined as the ratio of the lateral size to the thickness, which represents the shorter dimension of the needle-like particles (lateral size/thickness). The number of particles in an area of at least 15,000 μm^2^ was also counted and averaged. Energy-dispersive X-ray (EDX; Quantum75, Bruker) mapping images of Product-A were also obtained. The sample transparency and valence of vanadium ions were assessed by transmission spectroscopy (V670, JASCO; equipped with an absolute reflectance measurement unit (ARSN-733, JASCO)) using ca. 1.5-mm-thick glass specimens. The 670–690 nm range for some of the spectra was instrumentally derived.^6−8^ The mechanical properties of Product-H after crystallization were examined by Vickers hardness tests (HMVG20, Shimadzu) with a 1 kgf load and a 15 s holding time. Vickers hardness values were estimated using the average of twenty indentations.

## Results and Discussion

**Figure 1:**
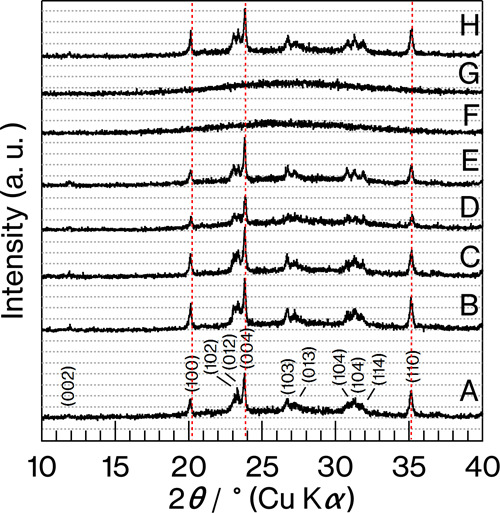
XRD patterns for Product-A to H.

Figure 1 shows XRD patterns for the glass specimens. The profiles for Product-A to -E and -H, all of which fit well with those reported in our previous studies,^5−9^ exhibit reflections attributed to metastable CaAl_2_Si_2_O_8_ crystals with layers stacked in the *c*-axis direction.^19^ In addition, reflections attributed to calcium vanadate^20^ were absent in all the profiles. Compared with the profile for Product-A, the intensity of the (004) reflection relative to other reflections is slightly increased for Product-B and -C. A slight increase in the (004) intensity is also observed for Product-E compared with Product-D. In general, the stacking order of layered crystals are varied in terms of crystallinity, stacking order, number, size, and aspect ratio for the layers.^21−23^ Meanwhile, the profiles for Product-F and -G show a halo pattern, indicating that no crystallization occurred.

**Figure 2:**
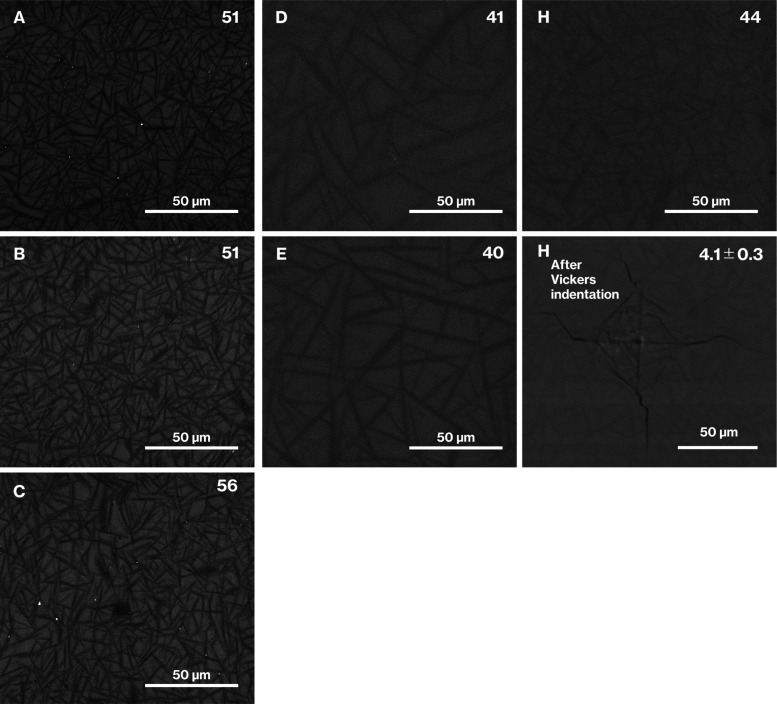
SEM images of Product-A to -E and -H in addition to Product-H after Vickers indentation. Volume fraction (vol %) or Vickers hardness are denoted in the upper-right corners of the SEM images.

Figure 2 shows SEM images of the galss specimens. All the SEM images display a cross-section of a house-of-cards structure similar to those reported previously.^4−9^ In addition, the volume fraction did not greatly differ between Product-A, -B, and -C. The difference between Product-D and -E also followed a similar trend. Although needle-like particles in Product-H are not clearly observed compared with Product-A to -E, the Vickers hardness for Product-H is 4.1 ± 0.3, which is close to the value of 4.2 ± 0.3 obtained for CAS GC-H, which has been often denoted as a standard in our previous reports.^4−8^

**Figure 3:**
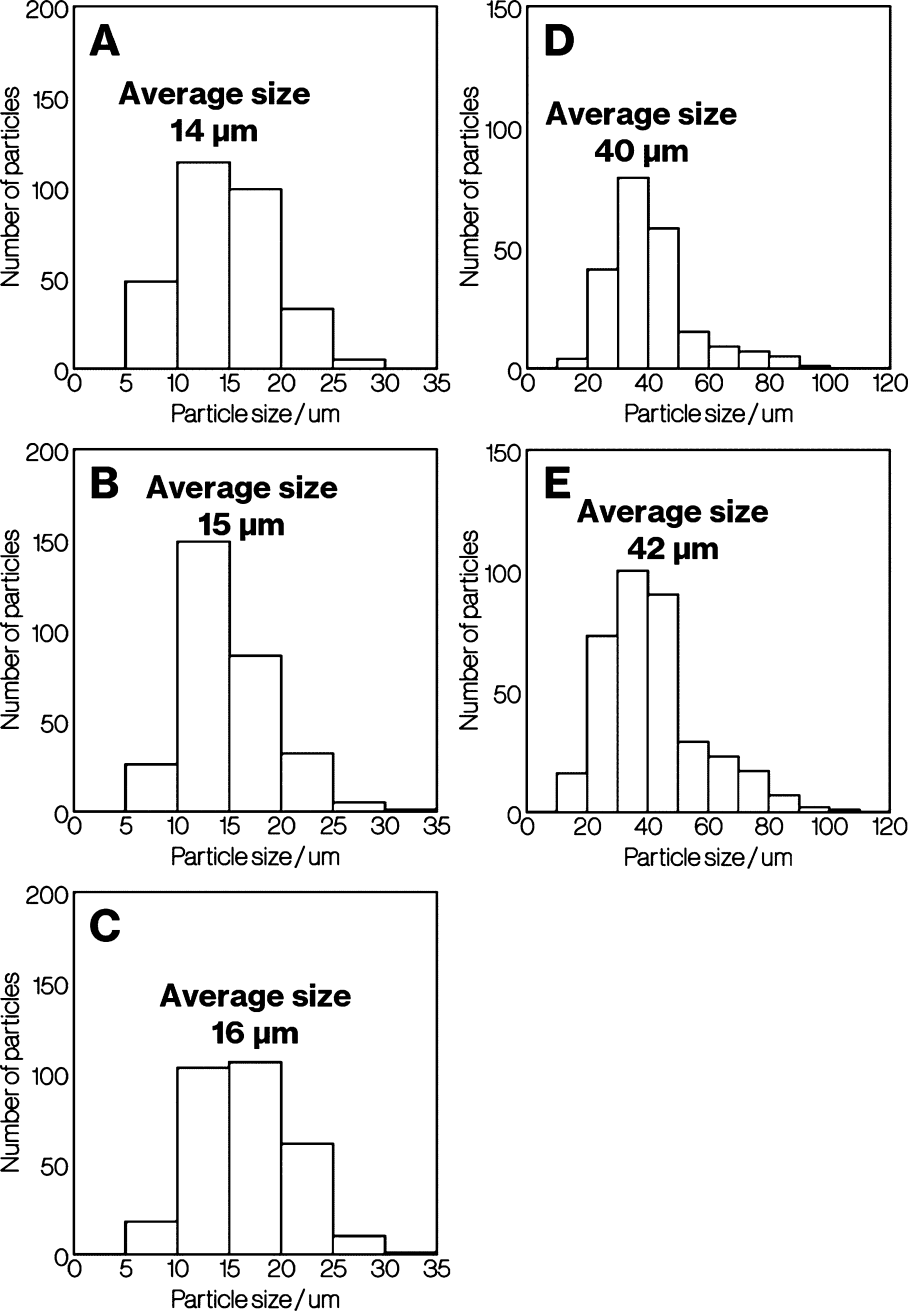
Particle size distributions for Product-A to -E.

Figure 3 shows particle size distributions for Product-A to -E. The deviations for Product-A to E are 4.3, 4.5, 4.2, 14, and 17, respectively. The distributions for Product-A and -C seem essentially the same, whereas the particle size increases slightly in the order of Product-A to -C. The relationship between Product-D and -E behaves is similar to that between Product-A and -C.

**Figure 4:**
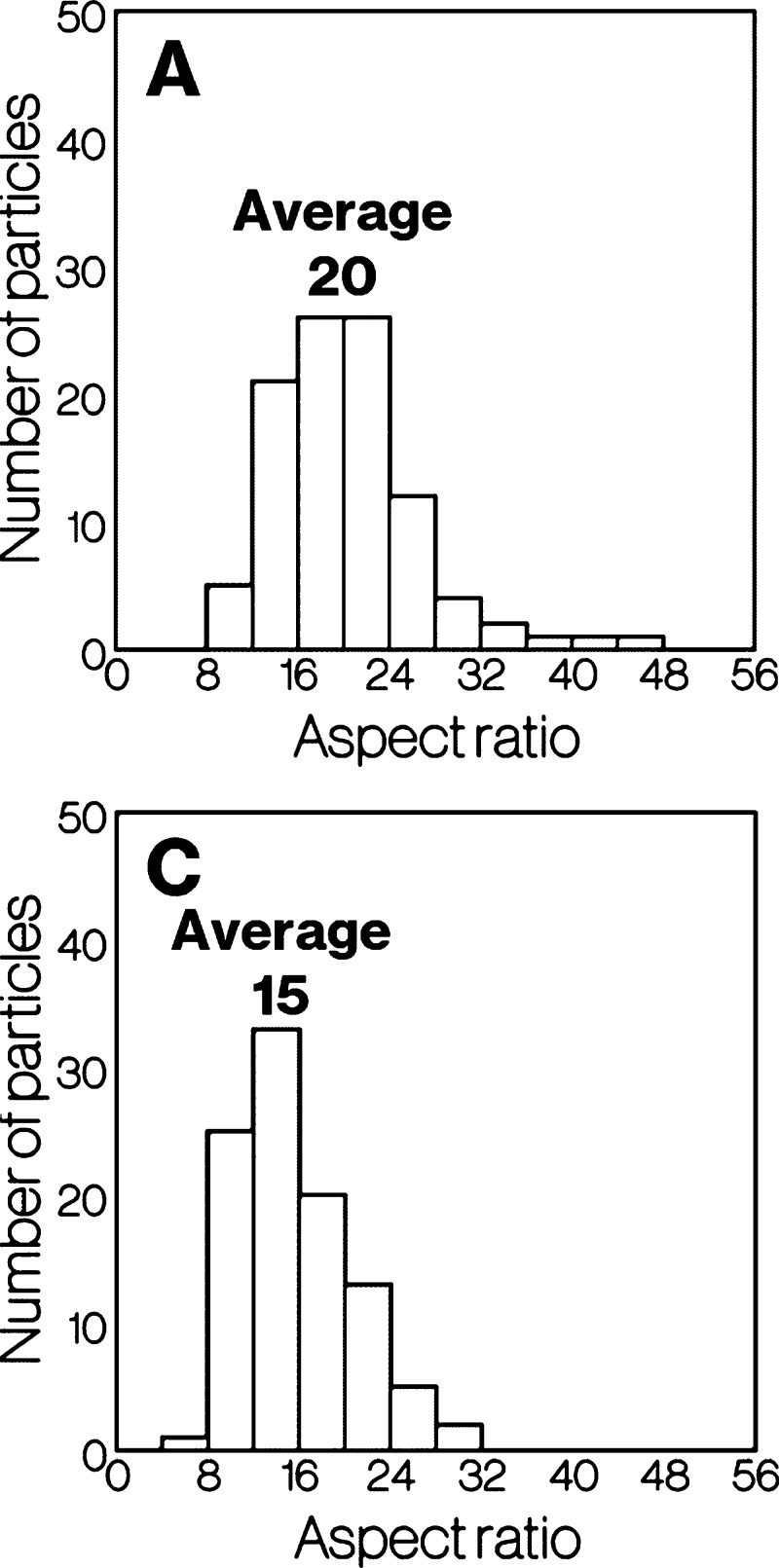
Distribution of aspect ratio for Product-A and -C.

Figure 4 shows the distributions of the aspect ratios for Product-A and -C. The deviations for Product-A and -C are 4.9 and 6.4, respectively. Although an arbitrary cross-section of a house-of-cards structure contains diagonal cross-sections of platy particles, the difference in the thickness of platy particles can be roughly estimated. The results indicate that Product-A has a larger aspect ratio than Product-C. The shorter dimensions of the relatively small needle-like particles in Product-D and -E are unfortunately ambiguous, while the needle-like particles in Product-D appear thinner than those in Product-E (Figure 2).

**Figure 5:**
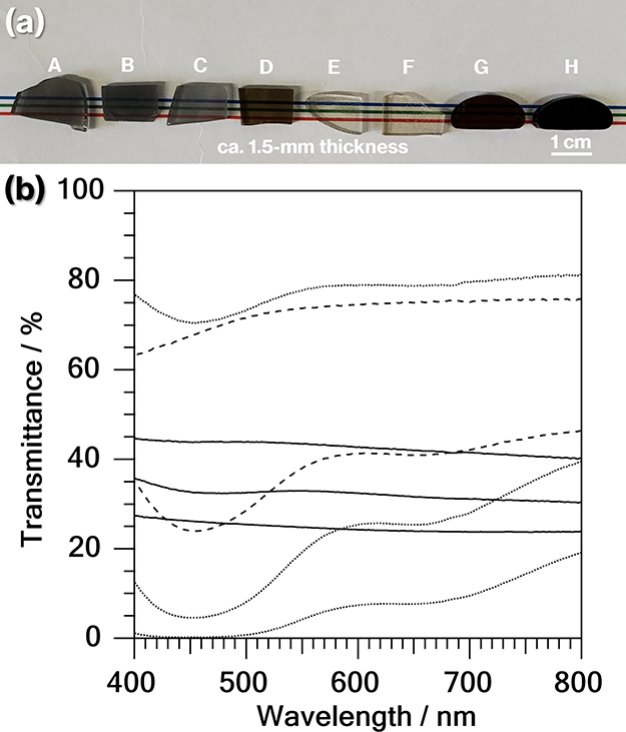
(a) Photograph and (b) transmission spectra of parent glass for Product-A to -C (middle, lower, and upper solid lines), Product-D and -E (bottom and top dashed lines), and Product-F to -H (dotted lines from top to bottom).

Figure 5 shows the photographs and transmission spectra of the parent glasses for Product-A to -H. As can be seen, the parent glass for Product-C is more transparent than that for Product-B. This is because MoO_3_ reduction occurs to a lesser extent during glass melting due to the smaller carbon content of Product-C than Product-B (see Table 1). The black coloration of Product-B and -C is attributed to the presence of metallic Mo particles, a result that matches well with those reported previously.^4−9^ Compared with the spectrum of the parent glass for Product-B and -C, the spectrum of the parent glass for Product-E shows a decrease in transmittance in the 400–500 nm range, which can likely be attributed to molybdenum and its oxide clusters.^24−27^ The black coloration of bulk metallic Mo is thus unlikely to have been generated by a decrease in carbon content in the raw materials from 0.40 wt % C for Product-B and -C, as well as CAS GC-H in our previous reports,^4−9^ to 0.080 wt % for Product-E, although a detailed discussion is beyond the scope of this work and will be reported in another study. Meanwhile, the V_2_O_3_-containing products (Product-A, -D, -F, -G, and -H) show light or dark red coloration and a decrease in transmittance in the 400–500 nm and/or 600–700 nm ranges that can be attributed to the presence of V^3+^ and V^4+^.^28^ In addition, the red coloration is darker in the order Product-F, -D, -G, and -H. In a previous study,^22^ light and dark red colorations due to V^3+^ and V^4+^ were obtained for aluminoborosilicate glass with a V_2_O_5_ content in the 1–5 mol % range, all of which also contained V^5+^. The X-ray photoelectron spectra of these glass specimens did not show evidence of a V^0^ chemical shift.^28,29^ Meanwhile, the white spots attributed to metallic particles^6,11^ and observed in the SEM images of Product-A to- C were absent for Product-H (Figure 2). Therefore, V^0^ is unlikely to be present in the glass specimens containing V_2_O_3_. Notably, in the present study, the V_2_O_3_ content in these glass specimens is in the 0.02–0.4 mol % range that is hardly detected in the characteristics.

Based on the results described above, precipitation of metastable CaAl_2_Si_2_O_8_ in the glassy phase is evident for Product-A to -E and -H (Figures 1 and 2). Because Product-F and -G, in which 0.052–0.52 wt % V_2_O_3_ was present but MoO_3_ was absent, did not show crystallization (Figure 1), the V_2_O_3_ content in the range of 0.052–0.21 wt % did not act as a nucleation agent for the precipitation of metastable CaAl_2_Si_2_O_8_. Thus, the V_2_O_3_ content in the range of 0.052–0.21 wt % in Product-A and -D did not directly affect the nucleation process for metastable CaAl_2_Si_2_O_8_. Therefore, the crystallization of metastable CaAl_2_Si_2_O_8_ in Product-A proceeded in the presence of a relatively smaller content of vanadium oxide (Figures 1, 2, 5). According to the distributions of particle size and aspect ratio (Figures 3 and 4) as well as the SEM images (Figure 2), Product-A displays a slight decrease in particle size and thickness compared with Product-B and -C. The differences in particle size and thickness between Product-D and -E are similar to those between Product-A, -B, and -C (Figures 2–4). In addition, the intensity of the (004) reflection relative to other intensities for the vanadium oxide-containing glass specimens (Product-A and -D) is slightly weaker than those for the glass specimens without vanadium oxide (Product-B, -C, and -E) (Figure 1). Therefore, the crystal growth of metastable CaAl_2_Si_2_O_8_ in the CAS glass was retarded in the *c*-axis direction^19^ with the preservation of the lateral size of platy particles to a certain extent in the presence of a relatively small vanadium oxide content, which unlikely depends on the size of platy particles of metastable CaAl_2_Si_2_O_8_ (Figures 3 and 4). The feasible mechanisms are discussed below.

**Figure 6:**
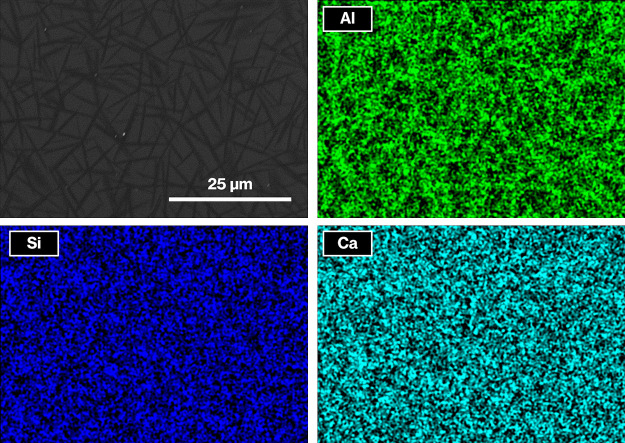
SEM image of Product-A (upper left) and EDX mappings for Al, Si, and Ca.

In previous studies, both vanadium (V^5+^ and V^4+^) and molybdenum oxides were present in the glass network forming region^12−16,28^ and were less rigid compared with alumina and silica.^13,29^ Although V^3+^ acting as a network modifier was proposed in the previous studies,^15,16^ on the basis of vanadium structural units surrounded by oxygens^27^ and the presence of tetragonal molybdenum oxide near glass network modifiers,^13^ V^3+^ units might be present near glass network modifiers similar to molybdenum oxide units. It is well-known that alumina acts as a network former. In the present study, the EDX mappings of Al, Si, and Ca of Product-A (Figure 6) are not clear, whereas the needle-like appearance is mapped on Al relative to Si and Ca. Although no detectable differences in EDX mappings for Al were observed between Product-A to -E (data not shown), alumina in the present CAS glass composition is relatively consumable by the crystallization of metastable CaAl_2_Si_2_O_8._ Alumina incorporation in the layered crystals is thus likely to be greater on the layered surfaces than at the edge surfaces because it is evident that the contact area of the layered surfaces with the glassy phase is significantly greater. In addition, the Al ratio in the region around the crystalline phase of Product-A and -D is made smaller by the addition of V_2_O_3_. Therefore, given a decrease in particle size of platy particles by the addition of V_2_O_3_ as shown in Figure 3, crystal growth in the stacking direction of the aluminosilicate layers of metastable CaAl_2_Si_2_O_8_ is retarded relatively to the lateral direction, decreasing the thickness of the layered crystals. Thus, V_2_O_3_ acts as an additive to control the thickness and/or aspect ratio of platy particles of metastable CaAl_2_Si_2_O_8_. Judging from the SEM images of CAS GC-Hs in our previous reports, relatively thin platy particles were observed for CAS GC-Hs prepared under an oxidizing atmosphere.^7^ Dark coloration due to metallic Mo and W particles was less in these CAS GC-Hs.^7^ Therefore, molybdenum and tungsten oxides generated by the oxidation of metallic Mo and W, whose amounts in the glass specimens are less than 0.1 wt %,^7^ during glass melting could also affect the retardation of crystal growth in the stacking direction of metastable CaAl_2_Si_2_O_8_. Because the oxidation free energy of vanadium is lower than that of molybdenum,^11^ molybdenum oxide might be reduced by vanadium oxide. In our previous reports,^6,7^ a stronger reducing atmosphere at the glass melting stage showed a large size of nucleation agents for increasing crystal particle sizes. In addition, judging from SEM images in the previous report,^7^ CAS GC-H prepared using metallic Mo particles under a stronger reducing atmosphere showed an increase in thickness of platy particles of metastable CaAl_2_Si_2_O_8_. In the present study, such a tendency is not observed, suggesting that the reaction between vanadium and molybdenum oxides did not occur. In a previous report, it was proposed that an increase in the aspect ratio of platy particles in composite materials improved the mechanical properties.^30^ In addition, larger platy particles in CAS GC-Hs relative to those in the present study have been obtained by various techniques to control the size of the particles.^6−9^ Furthermore, subsurface cracks introduced into CAS-GC-H that dominate the mechanical properties were recently analyzed by X-ray computed tomography.^10^ Therefore, a further study will be required to clarify the methods for controlling the aspect ratio of platy particles in glass for which other glass network formers such as boron and germanium oxides and^31^ their amounts to be added to parent glasses could be also feasible candidates.

Notably, a relatively large amount of V_2_O_3_ in the CAS glass induced the crystallization of metastable CaAl_2_Si_2_O_8_ based on the XRD pattern and SEM images before and after Vickers indentation of Product-H. The crystalline phase has never before appeared without using metallic Mo or W particles as nucleation agents.^4−9^ Given the possibility of the absence of metallic V in Product-H as discussed above, this study demonstrated for the first time the crystallization of metastable CaAl_2_Si_2_O_8_ crystals in CAS glass by the addition of an oxide, whereas vanadium oxide has already been used as a nucleation agent for the precipitation of barium celsian in BaO–Al_2_O_3_–SiO_2_ glass in which MoO_3_ was also useful for nucleation.^31^ Thus, other oxides^31^ that have been used for nucleation of crystals in glass with other compositions are possible candidates. Therefore, the detailed mechanisms for the crystallization of RAl_2_Si_2_O_8_ (R represents Ca, Sr, and Ba)^32,33^ are warranted to further investigation. In this trial, an increase in the crystallinity of CAS-GC-H, which will be analyzed by Rietveld refinement,^19,34^ by changing the composition,^34^ starting materials,^6,8^ and melting condition^7^ could be helpful for increasing the amount of vanadium oxide to be added, which exceeds the detection limits of characteristics. In this trial, the instrument using synchrotron^35^ could also be helpful. We will intend to pursue such studies in the future.

## Conclusions

We have demonstrated the effect of vanadium oxide on the crystallization of CAS glass to precipitate metastable CaAl_2_Si_2_O_8_, a layered crystal, using metallic Mo particles as nucleation agents. The CAS GC-Hs containing 0.062–0.12 wt % vanadium oxide displayed platy particles of metastable CaAl_2_Si_2_O_8_ with a relatively large aspect ratio compared with CAS GC-Hs prepared without the addition of vanadium oxide. Because no crystallization occurred in CAS glass with 0.062–0.12 wt % vanadium oxide in the absence of metallic Mo particles, it appeared that vanadium oxide acted as an additive to retard crystallization in the stacking direction of the aluminosilicate layers of metastable CaAl_2_Si_2_O_8_. Additionally, although CAS GC-Hs have never previously been obtained without using metallic Mo or W particles,^5−9^ a CAS glass with a higher vanadium oxide content (1.0 wt %) exhibited the crystallization of metastable CaAl_2_Si_2_O_8_. Thus, vanadium oxide is the first oxide to act as a nucleation agent for the precipitation of metastable CaAl_2_Si_2_O_8_ in CAS glass. Therefore, these results pave the way to the control of the aspect ratio of layered crystals in glass and have the potential to elucidate the mechanism for crystallization in CAS glass.
